# Trajectories of symptom burden and associated factors in patients with liver cirrhosis: a prospective study

**DOI:** 10.3389/fmed.2026.1792795

**Published:** 2026-03-30

**Authors:** Qian Liu, Chengde Su, Qianqian Zhu, Mingdan Li, Yali Xu, Ying Zhang, Xinyi Zhang, Yawen Luo, Ping Yang

**Affiliations:** 1 Department of Nursing, Affiliated Hospital of Zunyi Medical University, Zunyi, China; 2 Department of Infectious Diseases, Affiliated Hospital of Zunyi Medical University, Zunyi, China; 3 School of Nursing, Zunyi Medical University, Zunyi, China

**Keywords:** cirrhosis, symptom burden, latent class growth model, longitudinal study, influencing factors, trajectories

## Abstract

**Background:**

Liver cirrhosis is frequently accompanied by multiple concurrent symptoms that cluster together, resulting in a high symptom burden and impairments in patients’ quality of life and functional status. However, most existing studies rely on cross-sectional or static observational designs, which limit the exploration of the temporal sequence and potential causal relationships among symptoms. This study aimed to identify trajectories of symptom burden in patients with liver cirrhosis and to explore predictors of trajectory heterogeneity using latent class growth modeling (LCGM).

**Methods:**

A prospective longitudinal study was conducted from August 2023 to June 2024. A total of 223 hospitalized patients with liver cirrhosis were recruited from the Department of Infectious Diseases, a tertiary hospital in Zunyi, Guizhou Province, China, using convenience sampling. Data were collected at four time points—baseline (T₀, within 1 day of admission), 1 month (T₁), 3 months (T₂), and 6 months (T₃) after discharge—using a general information questionnaire and the Chinese version of the Memory Symptom Assessment Scale. Data were analyzed using SPSS 29.0 and Mplus 8.3.

**Results:**

Three distinct trajectories of symptom burden were identified: a high symptom-level risk group (C1, 12.56%), a moderate symptom-level decreasing group (C2, 36.32%), and a low symptom-level decreasing group (C3, 51.12%). Multinomial logistic regression analysis indicated that sex, average daily exercise duration, and Child–Pugh classification were significant predictors of trajectory membership (*p* < 0.05). Notably, one month after discharge appeared to be a critical turning point for patients transitioning to the high symptom-level risk trajectory.

**Conclusion:**

The trajectories of symptom burden in patients with liver cirrhosis exhibit significant heterogeneity. Female sex, average daily exercise duration of less than 1 h, and Child–Pugh class C were associated with unfavorable symptom burden trajectories. Early identification of high-risk patients may help healthcare providers implement timely and targeted interventions to reduce symptom burden and improve clinical outcomes and quality of life.

## Introduction

Cirrhosis is a major global public health issue, and its disease burden continues to rise due to the increasing prevalence of key etiological factors such as alcohol abuse, metabolic-associated fatty liver disease, and viral hepatitis (1–4). Characterized by an insidious onset and prolonged disease course, cirrhosis is often accompanied by multiple complications, leading to poor patient prognosis (5, 6). Studies have reported that patients with liver cirrhosis commonly experience multiple concurrent symptoms, with pain, abdominal distension, and fatigue as core components. These symptom clusters contribute to an increased symptom burden, impair patients’ quality of life and functional status, and may lead to treatment discontinuation and excessive use of healthcare resources (7, 8). Therefore, given the dynamic evolution of symptoms and their significant impact on disease prognosis (9, 10), systematically investigating the underlying mechanisms and influencing factors of changes in symptom burden in patients with liver cirrhosis is of critical importance. Such research can provide a foundation for establishing a scientifically grounded dynamic monitoring system and holds key implications for improving clinical outcomes in this population.

Most existing studies on symptom clusters have relied on cross-sectional designs, which limits the ability to fully capture the dynamic evolution of symptoms and may result in potentially ineffective clinical interventions (11). Evidence suggests that the onset or exacerbation of symptom clusters in patients with liver cirrhosis can signal disease progression or deterioration and directly prompt patients to seek medical attention (8). Furthermore, due to disease progression and unpredictable symptom fluctuations, these patients experience increased demands for self-management of their symptoms (12). These findings highlight the importance of establishing a longitudinal, dynamic assessment system for symptom burden and implementing targeted, stratified interventions to mitigate the impact of symptom clusters on disease progression and quality of life. However, the temporal patterns and characteristics of overall symptom changes in patients with liver cirrhosis remain insufficiently understood and warrant further investigation.

Due to individual differences in experiences and characteristics among patients with liver cirrhosis, some patients may exhibit high scores on certain specific symptoms while maintaining a relatively low symptom burden. This phenomenon may obscure the specificity of individual symptoms and overlook inter-individual variability, limiting the ability to investigate longitudinal changes in symptom subgroups (13). Evidence suggests that distinguishing heterogeneous symptom subgroups based on individual symptom trajectories may be a critical step for effective symptom management (14). Therefore, elucidating the substantive and variable nature of symptom patterns in patients with liver cirrhosis is essential for the identification and management of symptom subgroups. To support the development of comprehensive and evidence-based symptom management strategies, researchers have increasingly turned to analytical approaches that adopt an individual-centered perspective. Latent Class Growth Modeling (LCGM) is one such individual-centered method; by identifying latent subgroups with similar symptom evolution patterns, it not only more accurately reflects clinical heterogeneity but also provides a scientific basis for personalized interventions (15).

Therefore, this study is the first to apply latent class growth modeling (LCGM) to examine the trajectories of symptom burden in patients with liver cirrhosis and to identify their influencing factors. By overcoming the limitations of traditional cross-sectional designs, this approach provides a theoretical basis for developing individualized, dynamic symptom management strategies at early stages for patients with liver cirrhosis.

## Methods

### Study design and participants

From August 2023 to June 2024, a convenience sampling method was used to recruit patients with cirrhosis who were hospitalized in the Department of Infectious Diseases of a tertiary general hospital in Zunyi, Guizhou Province, China. Data were collected prospectively during this period.

Inclusion criteria:

1. The primary medical diagnosis met the *Guidelines for the Diagnosis and Treatment of Cirrhosis (2019 edition)* (16);
2. Age ≥ 18 years;
3. No other life-threatening comorbidities, such as acute heart failure or renal failure;
4. Conscious, with basic communication ability.

Exclusion criteria:

1. Presence of other severe chronic diseases, such as asthma or tuberculosis;
2. Presence of severe primary hematologic disorders or malignancies;
3. History of hepatocellular carcinoma surgery or liver transplantation;
4. Presence of psychological disorders, psychiatric illness, or chronic encephalopathy.

Withdrawal criteria:

1. Death during hospitalization or within 6 months after discharge;
2. Diagnosis of hepatocellular carcinoma or liver transplantation within 6 months after discharge;
3. Loss to follow-up for two consecutive assessments.

### Data collection

Data were collected from patients with cirrhosis at four specific time points: on the first day of hospital admission (T₀), and at 1 month (T₁), 3 months (T₂), and 6 months (T₃) after discharge.

At T₀, written informed consent was obtained from each participant prior to the survey. The researchers provided standardized instructions to explain the purpose and content of the questionnaires. For participants who were unable to read, the researchers read the questions and possible responses aloud in a neutral tone, allowing the patients or their family members to select the appropriate answers. The researchers assisted in completing the questionnaire when necessary. All questionnaires were collected immediately after completion, checked for completeness, and any missing or incorrect information was corrected in real time.

At T₁–T₃, follow-up data were collected through outpatient visits, telephone interviews, or the WeChat platform. A total of 223 valid questionnaires were obtained. All data were double-checked and entered independently by two researchers to ensure accuracy and reliability. The measurement instruments used at each time point (T₀–T₃) are presented in Table 1.

**Table 1:** Data collection scale for patients with cirrhosis.

Measurement time point	General information questionnaire	Chinese version of Memorial Symptom Assessment Scale
T_0_	√	√
T_1_		√
T_2_		√
T_3_		√

T_0_, Day 1 of hospital admission; T_1_, 1 month after discharge; T_2_, 3 months after discharge; T_3_, 6 months after discharge.

### Measurement instruments

#### General demographic and clinical information questionnaire

A self-designed questionnaire developed by the research team was used to collect general demographic and clinical information. The questionnaire consisted of two main sections: Demographic characteristics: age, gender, ethnicity, educational level, place of residence (rural or urban), average monthly household income, marital status, average daily exercise duration, drinking history, and smoking history. Drinking history was defined as an average daily alcohol consumption of >30 g for men and >20 g for women (17). Smoking history was defined as a smoking index (number of cigarettes per day × years of smoking) ≥ 200 (18). Clinical disease-related information: duration of cirrhosis (months/years), stage of cirrhosis (compensated or decompensated), etiology, Child–Pugh classification, presence of ascites, and complications (e.g., hypersplenism, hypoproteinemia, electrolyte imbalance, spontaneous peritonitis, esophagogastric varices, portal hypertension, hepatorenal syndrome, etc.), as well as readmission within 30 days and length of hospital stay.

#### Chinese version of the Memorial Symptom Assessment Scale (MSAS)

The Memorial Symptom Assessment Scale (MSAS) was developed by the Memorial Sloan-Kettering Cancer Center in the United States and is currently one of the most widely used instruments internationally for assessing patients’ symptom experiences. It is a multidimensional self-report scale that evaluates various physical and psychological symptoms. The MSAS consists of four subscales—*physical symptoms, psychological symptoms, global distress index,* and *total MSAS score*—with a total of 32 items. Among these, 24 items assess the occurrence, frequency, severity, and distress level of symptoms experienced during illness and treatment, while the remaining 8 items assess the occurrence, severity, and distress level of symptoms. Higher scores indicate more severe symptom experiences (19) The Chinese version of the MSAS was translated and validated by Fu et al. (20), demonstrating good psychometric properties, with Cronbach’s *α* ranging from 0.79 to 0.97 and content validity of 0.94. The total MSAS score is calculated as the mean of the 32 symptom items, with higher scores on each subscale indicating a greater symptom burden.

#### Supplementary items and reliability and validity of the Chinese version of the memorial symptom assessment scale

A pilot survey was conducted using a convenience sample of 30 hospitalized patients with liver cirrhosis from the Department of Infectious Diseases at a tertiary hospital in Zunyi, Guizhou Province, China, to evaluate the feasibility of questionnaire completion and further refine the scale’s content and items. Considering the disease-specific characteristics of liver cirrhosis, and based on literature review and pilot testing, four additional symptom items were incorporated into the Chinese version of the MSAS: bitter taste in the mouth, muscle cramps, dark urine, and scleral icterus (11, 21, 22). After adding these items, the Cronbach’s *α* coefficient of the revised Chinese version of the MSAS was 0.869, indicating good internal consistency and reliability of the modified scale.

#### Sample size

The sample size was calculated using the estimation method for survey studies in non-experimental research. PASS 15.0 software was employed, with a confidence level of 0.95, *α* = 0.05, and d = 0.07, based on the corresponding calculation formula.


$$n=\frac{Z\frac{\begin{array}{c} 2\\ \alpha \end{array}}{2}\pi (1-\pi )}{\delta^{2}}$$

Considering potential sample attrition during follow-up and assuming a response rate of 70–90%, the estimated sample size ranged from 126 to 165 participants. After accounting for an anticipated 20% loss to follow-up, the required sample size was adjusted to 152–198 participants. Furthermore, previous research (23) has indicated that when the Bayesian Information Criterion (BIC) is used as the primary criterion for model selection, the sample size should be at least 200. In this study, a total of 223 patients were included in the survey, which was higher than the minimum sample size requirement, indicating that the multivariate analysis of this study had basic statistical test power. In addition, for the small amount of missing data occurring during the longitudinal follow-up, this study adopted the multiple imputation method for standardized processing, and simultaneously used the complete case analysis method to conduct sensitivity verification. The results showed that there was no significant difference in the statistical results between the two methods (*p* > 0.05), ensuring data integrity and the reliability of the analysis.

### Statistical analysis

Data were managed and analyzed using SPSS 29.0 (IBM Corporation, Armonk, NY, USA) and Mplus 8.3. The specific methods were as follows:

1. *Descriptive and normality analyses*: Demographic characteristics and scale scores were first assessed for normality. Continuous variables with a normal distribution were expressed as mean ± standard deviation ( $\bar{x}$ ±SD) and analyzed using t-tests or ANOVA. Continuous variables not normally distributed were expressed as median (P_25_, P_75_) and analyzed using rank-sum tests. Categorical variables were presented as percentages and analyzed using the chi-square (χ^2^) test.
2. *Latent Class Growth Modeling (LCGM)*: Mplus 8.3 was used to establish latent class growth models to explore the trajectories of symptom changes among patients with cirrhosis. Model fit was evaluated using the following indices (24): Log Likelihood (LL), Akaike Information Criterion (AIC), Bayesian Information Criterion (BIC), and sample size-adjusted BIC (aBIC): Lower values indicate better model fit, with the model having the smallest BIC generally selected as the optimal model. Lo–Mendell–Rubin adjusted likelihood ratio test (LMR) and Bootstrapped likelihood ratio test (BLRT): A corresponding *p* < 0.05 indicates that a model with k classes fits better than a model with k–1 classes. Entropy: Used to evaluate the precision of individual classification into latent classes, ranging from 0 to 1. Higher values indicate more accurate classification, with Entropy ≥ 0.80 considered indicative of high classification accuracy. Information criteria (AIC, BIC, aBIC), entropy values, and BLRT were used to determine the best-fitting model. Multivariate logistic regression analysis was then employed to identify the predictors of different symptom cluster trajectories in patients with cirrhosis.
3. *Verification of LCGM assumptions and variable selection*: Before constructing the latent class growth model (LCGM) to analyze trajectories of symptom burden in patients with liver cirrhosis, the core assumptions of the model were verified. First, the independence of observations across participants was confirmed. Model residuals were assessed for normality and homogeneity of variance using the Shapiro–Wilk and Levene tests, respectively. Second, Little’s MCAR test indicated that missing follow-up data were not completely at random. Based on clinical characteristics, symptom scores were assumed to follow linear trajectories. Model fit was further evaluated using Akaike’s Information Criterion (AIC), Bayesian Information Criterion (BIC), and entropy values, confirming that the trajectory specification was appropriate. All assumptions were satisfied, ensuring the reliability of subsequent analyses. For variable selection, a systematic review of domestic and international literature was conducted, and candidate variables were identified based on clinical characteristics of patients with liver cirrhosis in China and consultation with experts in chronic liver disease. These variables were further refined through a pilot study involving 30 hospitalized patients with liver cirrhosis at the Department of Infectious Diseases of a tertiary Grade A hospital in Zunyi, China, to ensure the clinical relevance and appropriateness of the selected variables. This process ensured that the selected variables were both methodologically sound and clinically relevant.

### Ethical approval and informed consent

The study protocol was reviewed and approved by the Ethics Committee of the Affiliated Hospital of Zunyi Medical University (Approval No. KLLY-2023-064). Written informed consent was obtained from all participants prior to enrollment. All procedures were conducted in accordance with the principles of the Declaration of Helsinki and the relevant guidelines and regulations of the Ethics Committee of the Affiliated Hospital of Zunyi Medical University.

### Quality control

In this study, quality control was implemented throughout the entire research process. During the design phase, the study protocol was developed by reviewing domestic and international literature under the guidance of supervisors and the research team, appropriate survey instruments were selected, and clinical and nursing experts were consulted to optimize the study design. During the implementation phase, the research team established good patient–researcher relationships, obtained departmental support, and provided standardized training to researchers to ensure accurate questionnaire completion and consistent responses. During the data collection phase, researchers retrieved and checked all scales for completeness, promptly corrected any missing or erroneous items, and conducted follow-ups via outpatient visits, telephone, or WeChat, with participants who could not be reached for two consecutive follow-ups considered lost to follow-up. During the data processing and analysis phase, the data were examined for completeness and logical consistency, entered using a double-entry system with random checks to ensure accuracy, and analyzed strictly according to pre-specified statistical methods, thereby ensuring the scientific rigor and reliability of the study data.

## Results

### Follow-up overview

At baseline (T_0_), a total of 268 valid questionnaires were collected from hospitalized patients with liver cirrhosis. After a 6-month follow-up period following discharge, 223 patients successfully completed all follow-up assessments.

At the T_1_ time point (1 month post-discharge), 48 patients were rehospitalized, yielding a readmission rate of 21.52%. Between T_1_ and T_3_, 45 patients were lost to follow-up, corresponding to a loss rate of 16.79%. The reasons for loss to follow-up included loss of contact (*n* = 5), death (*n* = 28, mortality rate 10.45%), newly diagnosed liver cancer (*n* = 10, incidence 3.73%), and liver transplantation (*n* = 2).

**Figure 1:**
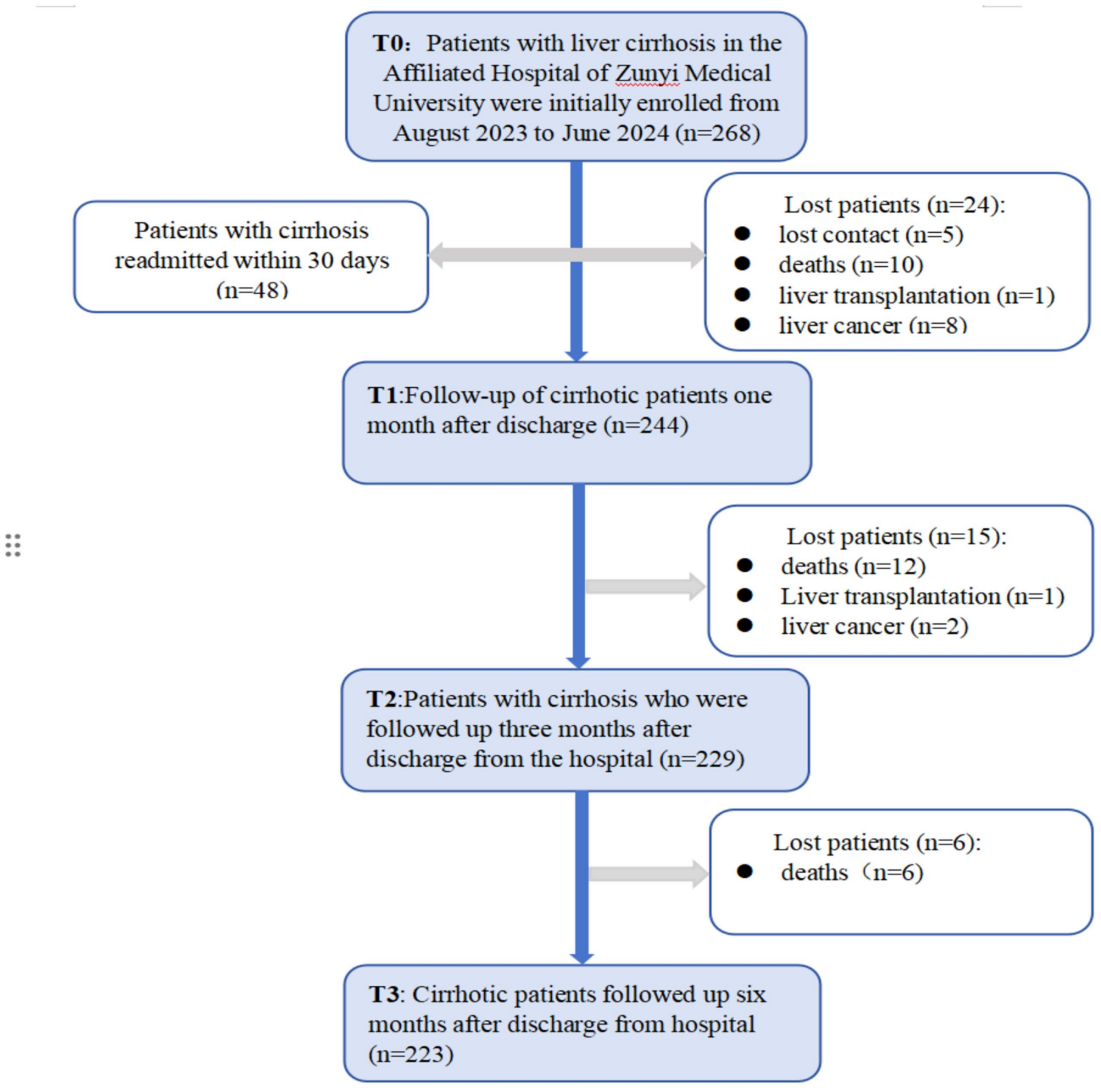
Flow chart for follow-up of patients with cirrhosis.

This prospective longitudinal study ultimately included 223 patients with liver cirrhosis who completed the entire follow-up process from baseline (T_0_) to 6 months post-discharge. The participant flow diagram is shown in Figure 1.

### Demographic and clinical characteristics of participants and comparison with lost-to-follow-up patients

Among the 223 patients with liver cirrhosis who completed the follow-up, the mean age was 52.51 ± 11.24 years, with the majority aged 45–59 years (50.20%). Of these, 158 were male (70.90%) and 65 were female (29.10%). The highest proportion of participants had completed junior high school or secondary vocational education (43.90%). Regarding medical insurance type, urban resident medical insurance was most common (77.60%). In terms of average monthly household income, 39.00% of patients reported an income of ≥418 USD. The majority of participants lived in rural areas (80.30%), while 19.70% resided in urban or city settings. A total of 138 patients (61.90%) had a history of smoking, and 132 patients (59.20%) reported a history of alcohol consumption. Additionally, 46.60% of patients engaged in ≤0.5 h of physical activity per day. No statistically significant differences were observed in demographic or clinical variables between the 223 patients who completed follow-up and the 45 patients lost to follow-up (*p* > 0.05), indicating baseline comparability between groups (Table 2).

**Table 2:** General characteristics of participants and analysis of lost-to-follow-up patients (*n* = 268).

Variable		Non-lost patients (*n* = 223)	Lost patients (*n* = 45)	Test statistic	*p*-value
Gender	Male	158 (70.90%)	30 (66.70%)	0.313	0.576
	Female	65 (29.10%)	15 (33.30%)		
Age (years)	18–44	48 (21.50%)	8 (17.8%)	1.644	0.440
	45–59	112 (50.20%)	20 (44.4%)		
	≥60	63 (28.30%)	17 (37.80%)		
Education	Illiteracy	22 (9.90%)	5 (11.10%)	0.842^a^	0.933
	Elementary school	67 (30.00%)	13 (28.90%)		
	Junior High School	98 (43.90%)	21 (46.70%)		
	High School/Junior College	29 (13.00%)	4 (8.90%)		
	College/University and above	7 (3.10%)	2 (4.40%)		
Marital status	Unmarried	7 (3.10%)	42 (93.30%)	0.296^a^	0.862
	Married	209 (93.70%)	1 (2.20%)		
	Divorced/Widowed	7 (3.10%)	2 (4.40%)		
Ethnicity	Han	198 (88.80%)	40 (88.90%)	0.000	0.985
	Minority	25 (11.20%)	5 (11.10%)		
Types of medical payments	Fully self-funded	19 (8.50%)	2 (4.40%)	4.372	0.112
	Urban Resident Medical Insurance	173 (77.60%)	41 (91.10%)		
	Employee Medical Insurance	31 (13.90%)	2 (4.40%)		
Average monthly income of family members (USD)	≤140	84 (37.70%)	20 (44.0%)	0.780	0.677
	140–418	52 (23.30%)	10 (22.20%)		
	≥418	87 (39.00%)	15 (33.30%)		
Residence	Towns/Cities	44 (19.70%)	32 (71.10%)	1.875	0.171
	Rural	179 (80.30%)	13 (28.90%)		
Smoking history	Yes	138 (61.90%)	28 (62.20%)	0.002	0.966
	No	85 (38.10%)	17 (37.80%)		
Alcohol history	Yes	132 (59.20%)	24 (53.3%)	0.528	0.467
	No	91 (40.80%)	21 (46.70%)		
Average daily exercise time (h/d)	≤0.5	104 (46.60%)	25 (55.60%)	5.909	0.052
	0.5–1	56 (25.10%)	15 (33.30%)		
	≥1	63 (28.30%)	5 (11.1%)		

USD, United States Dollar (CNY/USD: 7.1705); ^a^indicates Fisher’s exact test.

### Disease characteristics of participants and comparison with lost-to-follow-up patients

Among the 223 patients with liver cirrhosis who completed the follow-up, 109 cases (48.90%) were attributed to hepatitis B virus infection, followed by 45 cases (20.20%) of alcoholic cirrhosis. There were 64 patients (28.70%) in the compensated stage and 159 patients (71.30%) in the decompensated stage. A total of 119 patients (53.40%) had a disease duration of less than 6 months, and 37 patients (16.60%) had a disease duration of 1–3 years. The prevalence of ascites was 41.30%. Regarding precipitating factors for hospitalization, 173 patients (77.60%) had no identifiable trigger, while 14 patients (6.30%) were hospitalized due to self-discontinuation of medication. According to the Child–Pugh classification, 25.10% were classified as Grade A, 43.00% as Grade B, and 31.80% as Grade C. Additional detailed clinical characteristics are presented in Table 3. Comparative analysis between the 223 patients who completed follow-up and the 45 patients lost to follow-up revealed no significant differences in most disease characteristics (*p* > 0.05), except for stage of cirrhosis, disease duration, ascites, Child–Pugh classification, and presence of complications. This discrepancy may be attributed to the fact that death accounted for 62.22% of all losses to follow-up, and these clinical indicators are closely associated with the severity of disease progression. Further analysis indicated that lost-to-follow-up patients were more likely to present with advanced cirrhosis, ascites, disease duration ≥10 years, Child–Pugh Grade C, and multiple complications, all of which are linked to an increased risk of mortality, contributing to the higher attrition rate (Table 3).

**Table 3:** Clinical characteristics of patients with liver cirrhosis (*n* = 268).

Item	Variables	Non-lost patients (*n* = 223)	Lost patients (*n* = 45)	Test statistic	*P*-value
Disease etiology	Viral Hepatitis B	109 (48.90%)	21 (46.70%)	3.847^a^	0.427
	Alcoholic Hepatitis	45 (20.20%)	9 (20.00)%		
	Autoimmune Hepatitis	22 (9.90%)	4 (8.90%)		
	Combination of two or more	27 (12.10%)	3 (6.70%)		
	Other	20 (9.00%)	8 (17.80%)		
Cirrhosis staging	Reparative period	64 (28.70%)	6 (13.3%)	4.582	0.032
	Decompensated period	159 (71.30)	39 (86.70%)		
Duration of illness (months/years)	≤6 months	119 (53.40%)	17 (37.8%)	13.341^a^	0.020
	6 months – 1 year	12 (5.40%)	2 (4.40%)		
	1–3 years	37 (16.60%)	3 (6.70%)		
	3–5 years	14 (6.30%)	6 (13.30%)		
	5–10 years	27 (12.10%)	11 (24.40%)		
	≥10 years	14 (6.30%)	6 (13.30%)		
Ascites	Yes	92 (41.30%)	27 (60.00%)	5.329	0.021
	No	131 (58.70%)	18 (40.00%)		
Predisposing factor	No apparent cause	173 (77.60%)	35 (77.80%)	6.543^a^	0.478
	Self-discontinuation	14 (6.30%)	1 (2.20%)		
	Not taking medication regularly	11 (4.90%)	1 (2.20%)		
	Not taking medication	6 (2.70%)	2 (4.40%)		
	Alcohol consumption	6 (2.70%)	1 (2.20%)		
	Combination of two or more	4 (1.80%)	0 (0.00%)		
	Self-administered unknown drugs	2 (0.90%)	1 (2.20%)		
	Other	7 (3.10%)	4 (8.90%)		
Child-Pugh	A	56 (25.10%)	4 (8.90%)	19.700	<0.001
	B	96 (43.00%)	11 (24.40%)		
	C	71 (31.80%)	30 (66.70%)		
Days of hospitalization (days)	≤10	132 (59.20%)	21 (46.7%)	3.970^a^	0.137
	10–20	71 (31.80%)	16 (35.6%)		
	≥20	20 (9.00%)	8 (17.8%)		
Complications	None	60 (26.90%)	5 (11.10%)	10.347	0.016
	Merge 1	61 (27.40%)	9 (20.00%)		
	Merge 2	58 (26.00%)	14 (31.10%)		
	Merge 3 or more	44 (19.70%)	17 (37.80%)		

^a^indicates Fisher’s exact test.

### Prevalence and distress levels of symptoms at four time points

Table 4 presents the prevalence and distress levels of symptoms among patients with liver cirrhosis at four time points. A total of 36 symptoms were assessed, and both their prevalence and distress levels varied over time. At baseline (T_0_), *difficulty falling asleep* had the highest prevalence, while *abdominal distension* caused the greatest level of distress. At T_1_, T_2_, and T_3_, *difficulty falling asleep* remained the symptom with the highest prevalence and distress level.

**Table 4:** Prevalence of T_0_–T_3_ symptoms and degree of distress in cirrhotic patients.

Symptoms	T_0_ (*n* = 223)		T_1_ (*n* = 223)		T_2_ (*n* = 223)		T_3_ (*n* = 223)	
	%	*M* (*P*_25_, P_75_)	%	*M* (*P*_25_, P_75_)	%	*M* (*P*_25_, P_75_)	%	*M* (*P*_25_, P_75_)
Difficulty sleeping	96.4	3.00 (2.00,4.00)	90.1	4.00 (3.00,4.00)	89.7	3.00 (2.00,4.00)	74.9	3.00 (1.00,4.00)
Anxiety	94.6	3.00 (2.00,3.00)	72.6	2.00 (0.00,3.00)	61.0	2.00 (0.00,3.00)	57.0	2.00 (0.00,3.00)
Generalized weakness	88.3	3.00 (2.00,4.00)	85.7	3.00 (2.00,4.00)	77.1	2.00 (1.00,3.00)	67.7	2.00 (0.00,3.00)
Loss of appetite	86.5	3.00 (2.00,4.00)	66.4	2.00 (0.00,4.00)	55.6	2.00 (0.00,3.00)	47.1	0.00 (0.00,3.00)
Loss of interest in sex	83.0	2.00 (1.00,3.00)	71.3	2.00 (0.00,2.00)	47.1	0.00 (0.00,2.00)	48.4	0.00 (0.00,2.00)
Difficulty concentrating	77.6	0.00 (1.00,2.00)	48.9	0.00 (0.00,2.00)	31.4	0.00 (0.00,2.00)	23.3	0.00 (0.00,0.00)
Not looking like yourself	70.9	2.00 (0.00,3.00)	18.8	0.00 (0.00,0.00)	17.5	0.00 (0.00,0.00)	17.9	0.00 (0.00,0.00)
Yellow urine	67.3	1.00 (0.00,3.00)	12.6	0.00 (0.00,0.00)	10.3	0.00 (0.00,0.00)	11.3	0.00 (0.00,0.00)
Feeling bloated	66.4	4.00 (0.00,4.00)	48.4	0.00 (0.00,3.00)	39.0	0.00 (0.00,2.00)	32.9	0.00 (0.00,2.00)
Feeling nervous	63.7	1.00 (0.00,2.00)	31.4	0.00 (0.00,2.00)	20.2	0.00 (0.00,0.00)	16.6	0.00 (0.00,0.00)
Yellowish sclera	63.2	1.00 (0.00,3.00)	14.8	0.00 (0.00,0.00)	9.9	0.00 (0.00,0.00)	11.2	0.00 (0.00,0.00)
Dry mouth	62.3	2.00 (0.00,3.00)	52.0	2.00 (0.00,3.00)	48.0	0.00 (0.00,3.00)	49.3	0.00 (0.00,3.00)
Muscle cramps	61.0	1.00 (0.00,3.00)	45.3	0.00 (0.00,1.00)	46.6	0.00 (0.00,2.00)	48.0	0.00 (0.00,1.00)
Feeling sad	60.1	1.00 (0.00,2.00)	13.5	0.00 (0.00,0.00)	4.5	0.00 (0.00,0.00)	2.2	0.00 (0.00,0.00)
Pain (abdominal pain)	59.6	1.00 (0.00,3.00)	30.0	0.00 (0.00,2.00)	17.0	0.00 (0.00,0.00)	11.7	0.00 (0.00,0.00)
Bitter mouth	57.8	2.00 (0.00,2.00)	50.2	2.00 (0.00,3.00)	47.1	0.00 (0.00,2.00)	47.1	0.00 (0.00,3.00)
Skin changes (hyperpigmentation, yellowing, petechiae ecchymosis)	57.8	1.00 (0.00,3.00)	36.3	0.00 (0.00,2.00)	32.7	0.00 (0.00,2.00)	32.9	0.00 (0.00,2.00)
Swelling of limbs	40.4	0.00 (0.00,3.00)	30.9	0.00 (0.00,2.00)	26.0	0.00 (0.00,2.00)	22.4	0.00 (0.00,0.00)
Itchy skin	39.9	0.00 (0.00,2.00)	23.3	0.00 (0.00,1.00)	13.5	0.00 (0.00,0.00)	12.1	0.00 (0.00,0.00)
Change in eating taste	39.5	0.00 (0.00,1.00)	22.9	0.00 (0.00,1.00)	18.4	0.00 (0.00,0.00)	16.1	0.00 (0.00,0.00)
Nausea	31.4	0.00 (0.00,2.00)	7.2	0.00 (0.00,0.00)	3.6	0.00 (0.00,0.00)	0.9	0.00 (0.00,0.00)
Shortness of breath	26.9	0.00 (0.00,0.00)	5.8	0.00 (0.00,0.00)	3.6	0.00 (0.00,0.00)	0.9	0.00 (0.00,0.00)
Coughing	25.6	0.00 (0.00,0.00)	8.5	0.00 (0.00,0.00)	5.8	0.00 (0.00,0.00)	2.7	0.00 (0.00,0.00)
Sleepiness	25.6	0.00 (0.00,0.00)	4.0	0.00 (0.00,0.00)	2.7	0.00 (0.00,0.00)	-	
Weight loss	25.1	0.00 (0.00,1.00)	10.3	0.00 (0.00,0.00)	5.4	0.00 (0.00,0.00)	5.4	0.00 (0.00,0.00)
Dizziness	21.1	0.00 (0.00,0.00)	5.8	0.00 (0.00,0.00)	2.7	0.00 (0.00,0.00)	0.9	0.00 (0.00,0.00)
Constipation	17.0	0.00 (0.00,0.00)	6.3	0.00 (0.00,0.00)	4.9	0.00 (0.00,0.00)	4.5	0.00 (0.00,0.00)
Difficulty urinating (decreased urine output)	17.0	0.00 (0.00,0.00)	13.5	0.00 (0.00,0.00)	13.0	0.00 (0.00,0.00)	13.9	0.00 (0.00,0.00)
Vomiting	14.8	0.00 (0.00,0.00)	2.7	0.00 (0.00,0.00)	2.2	0.00 (0.00,0.00)	0.9	0.00 (0.00,0.00)
Numbness and tingling in hands and feet	11.2	0.00 (0.00,0.00)	4.5	0.00 (0.00,0.00)	1.8	0.00 (0.00,0.00)	1.8	0.00 (0.00,0.00)
Diarrhea	10.8	0.00 (0.00,0.00)	3.1	0.00 (0.00,0.00)	0.4	0.00 (0.00,0.00)	0.4	0.00 (0.00,0.00)
Sweating	9.9	0.00 (0.00,0.00)	2.7	0.00 (0.00,0.00)	0.40	0.00 (0.00,0.00)	-	-
Irritability	9.9	0.00 (0.00,0.00)	0.9	0.00 (0.00,0.00)	-	-	-	-
Mouth ulcers	1.8	0.00 (0.00,0.00)	2.7	0.00 (0.00,0.00)	0.90	0.00 (0.00,0.00)	0.40	0.00 (0.00,0.00)
Difficulty swallowing	2.7	0.00 (0.00,0.00)	0.9	0.00 (0.00,0.00)	-	-	-	-
Hair loss	31.0	0.00 (0.00,0.00)	2.2	0.00 (0.00,0.00)	0.40	0.00 (0.00,0.00)	0.9	0.00 (0.00,0.00)

### Trajectories and heterogeneity of symptom burden across four time points

Latent class growth modeling (LCGM) was employed to classify symptom patterns across four time points and to explore heterogeneous trajectory classes of symptom burden among patients with liver cirrhosis. Models with one to four latent classes were sequentially fitted, and model fit indices were compared to determine the optimal trajectory model. The results showed that as the number of latent classes increased, the values of the log-likelihood (LL), Akaike Information Criterion (AIC), Bayesian Information Criterion (BIC), and adjusted BIC (aBIC) gradually decreased. When the number of latent classes was three, the model demonstrated satisfactory fit, with an entropy value greater than 0.80, and both the Lo–Mendell–Rubin likelihood ratio test (LMR) and the bootstrap likelihood ratio test (BLRT) reaching statistical significance (*p* < 0.05). Although the four-class model yielded a lower BIC compared with the three-class model, the Lo–Mendell–Rubin likelihood ratio test (LMR) was not significant, and the class probabilities were suboptimal, with the smallest subgroup accounting for only 5.58% of the sample. This small proportion limits the model’s ability to represent meaningful subgroups of symptom burden and reduces its clinical applicability for targeted interventions. The subgroup characteristics of the 4-class model overlapped substantially with those of the 3-class model, failing to yield a clinically distinct trajectory type, while adding unnecessary complexity to clinical stratification and intervention. Considering all fit indices and practical interpretability, the three-class model (C1, C2, and C3) was selected as the final LCGM solution. Detailed model fit indices are presented in Table 5.

**Table 5:** Analysis of latent class indicators for trajectories of symptom burden in 223 patients with liver cirrhosis.

Symptom model	LL	AIC	BIC	aBIC	Entropy	LMR	BLRT	Category probability (%)
Class 1	−4624.48	9246.95	9292.21	9266.86	-	-	-	-
Class 2	−4494.83	9011.66	9049.14	9014.28	0.85	0.027	<0.001	74.89/25.11
Class 3*	−4436.73	8901.46	8949.16	8904.79	0.87	0.003	<0.001	36.32/12.56/51.12
Class 4	−4427.67	8889.33	8947.25	8893.38	0.85	0.350	<0.001	5.58/47.98/12.55/34.08

*Denotes the best-fitting model; LL, Log Likelihood; AIC, Akaike Information Criterion; BIC, Bayesian Information Criterion; aBIC, Sample size-adjusted BIC; LMR, Lo–Mendell–Rubin; BLRT, Bootstrapped likelihood ratio test.

The membership probability matrix for the three latent classes (C1, C2, and C3) of symptom burden trajectories among patients with liver cirrhosis is presented in Table 6. As shown, the average posterior probability of assignment to each class exceeded 0.90, indicating that the three-class latent class growth model (LCGM) for symptom burden trajectories has high classification accuracy and reliability.

**Table 6:** Mean probability of each group of patients being assigned to one of the three categories in the three-group latent category growth model.

Symptom model	C1 (%)	C2 (%)	C3 (%)
C1	0.96	0.04	0.05
C2	0.02	0.93	0.00
C3	0.06	0.00	0.94

### Characteristics of trajectory classes of symptom burden

In this study, the trajectories of symptom burden were characterized using the intercepts, slopes, and other parameters derived from the selected latent class growth model (LCGM). Based on the features of each trajectory, symptom distress scores, and temporal patterns across the four assessment points, the three latent classes were subsequently labeled as follows:

1. Category 1 (C1): high symptom level–risk group

This category included 28 patients (12.56% of the total sample). The intercept and slope values were 154.67 and −23.99, respectively (*p* < 0.05). The total symptom scores remained at a high level across the four time points, with initial scores higher than those of the other categories. The symptom levels showed more pronounced fluctuations between T_1_ and T_3_ compared with Categories 2 and 3.

1. Category 2 (C2): moderate symptom level–declining group

This category included 81 patients (36.32% of the total sample). The intercept and slope values were 131.11 and −52.75, respectively (*p* < 0.05). The total symptom scores were at a moderate level across the four time points, lying between those of Categories 1 and 3. From T_0_ to T_2_, symptom scores showed a continuous declining trend, and from T_2_ to T_3_, they stabilized.

1. Category 3 (C3): low symptom level–declining group

This category included 114 patients (51.12% of the total sample). The intercept and slope values were 89.41 and −53.43, respectively (*p* < 0.05). The total symptom scores remained at a low level throughout the four time points, showing a steady decline from T_0_ to T_2_ and stabilization from T_2_ to T_3_.

**Figure 2:**
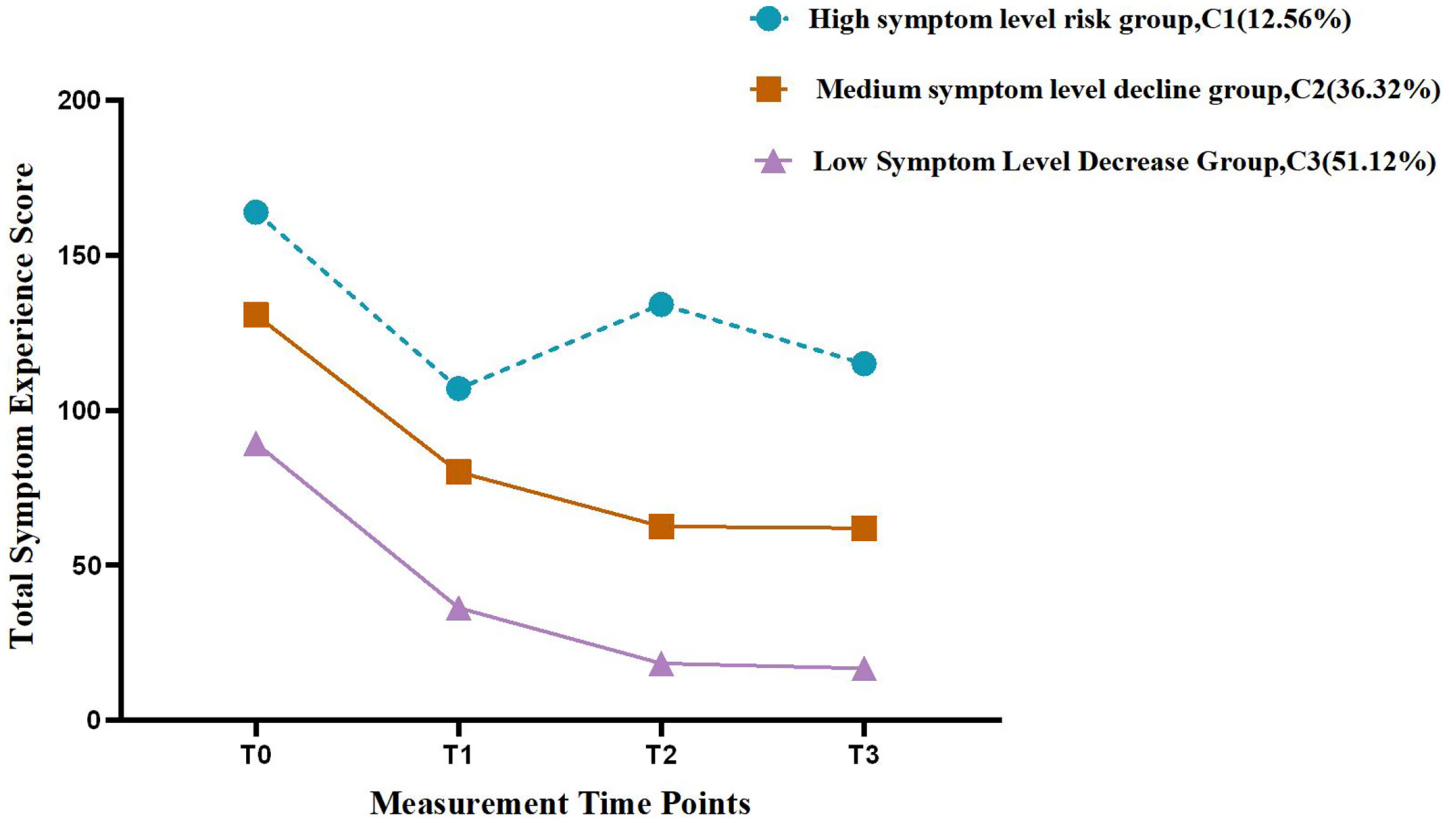
Symptom burden trajectories across different latent classes in patients with liver cirrhosis.

**Figure 3:**
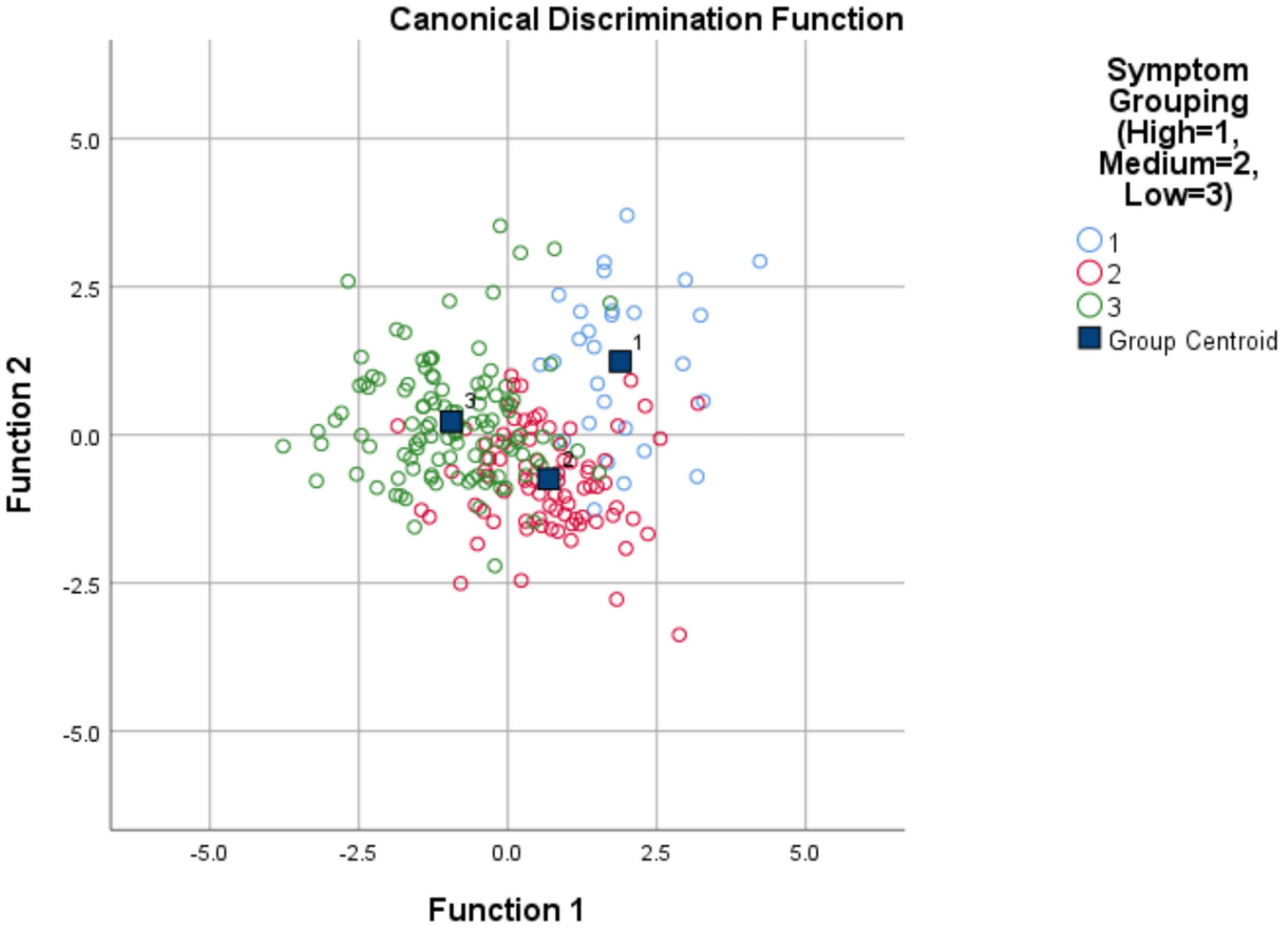
Discriminant analysis of the three-class latent class model for symptom burden trajectories in patients with liver cirrhosis (*n* = 223).

Detailed parameter estimates and trajectory patterns for each category are presented in Table 7 and Figure 2. Figure 3 visually demonstrates that the optimal model derived from the LCGM analysis exhibits good reliability and strong discriminative power.

**Table 7:** Parameter estimates of the LCGM for trajectories of symptom burden in patients with liver cirrhosis.

Symptom categories	Parameters	Estimated value	Standard error	*p*
C1				
Average value	Intercept	154.67	7.764	<0.001
	Slope	−23.99	7.589	0.002
C2				
Average value	Intercept	131.11	4.94	<0.001
	Slope	−52.75	5.82	<0.001
C3				
Average value	Intercept	89.41	5.31	<0.001
	Slope	−53.43	4.06	<0.001

### Analysis of factors associated with trajectories of symptom burden

To further investigate the heterogeneity of symptom burden trajectories in patients with liver cirrhosis and to identify and predict patterns of symptom change for early identification of high-risk patients, the three latent trajectory classes were treated as dependent variables. Demographic information and clinical characteristics collected at the baseline time point (T0) were included as independent variables to examine factors associated with trajectory membership.

### Univariate analysis of trajectory classes of symptom burden

The results of univariate analysis indicated that sex, age, educational level, per capita household income, average daily exercise time, stage of liver cirrhosis, presence of ascites, Child–Pugh classification, number of comorbidities, and length of hospital stay differed significantly across the trajectory classes of symptom burden among patients with liver cirrhosis (*p* < 0.05). Detailed results are presented in Table 8.

**Table 8:** Comparison of trajectory classes of symptom burden in patients with liver cirrhosis [*n* (%)].

Item	Variables	High symptom level risk group	Medium symptom level decline group	Low symptom level decrease group	$\chi^{2}$	*p*
*n*		28	81	114		
Gender	Male	14 (50.00)	56 (69.10)	88 (77.20)	8.230	0.016
	Female	14 (50.00)	25 (30.90)	26 (22.80)		
Age (years)	18–44	3 (10.70)	14 (17.30)	31 (27.20)	15.225	0.004
	44–59	12 (42.90)	37 (45.70)	63 (55.30)		
	≥60	13 (46.40)	30 (37.00)	20 (17.50)		
Education	Illiteracy	4 (14.30)	12 (14.80)	6 (5.30)	15.832^a^	0.047
	Elementary school	12 (42.90)	24 (29.60)	31 (27.20)		
	Junior High School	6 (21.40)	37 (45.70)	55 (48.20)		
	High School/Junior College	4 (14.30)	7 (8.60)	18 (15.80)		
	College/University and above	2 (7.10)	1 (1.20)	4 (3.50)		
Average monthly income of family members (USD)	≤140	15 (53.60)	38 (46.90)	31 (27.20)	13.706	0.008
	140–418	7 (25.00)	18 (22.20)	27 (23.70)		
	≥418	6 (21.40)	25 (30.90)	56 (49.10)		
Smoking history	Yes	15 (53.60)	45 (55.60)	78 (68.40)	4.261	0.119
	No	13 (10.70)	36 (44.40)	36 (31.60)		
Alcohol history	Yes	14 (50.00)	46 (56.80)	72 (63.20)	1.915	0.384
Alcohol history	Yes	14 (50.0)	35 (43.20)	42 (36.80)		
Residence	towns/cities	22 (78.60)	65 (80.20)	92 (80.70)	0.064	0.968
	rural	6 (21.40)	16 (19.80)	22 (19.30)		
Ethnicity	Han	26 (92.90)	71 (87.70)	101 (88.60)	0.575	0.750
	Minority	2 (3.10)	10 (12.30)	13 (11.40)		
Types of medical payments	Fully self-funded	2 (7.10)	6 (7.40)	11 (9.60)	0.429^a^	0.987
	Urban Resident Medical Insurance	22 (78.60)	64 (79.00)	87 (76.30)		
	Employee Medical Insurance	4 (14.30)	11 (13.60)	16 (14.00)		
Average daily Exercise time (h/d)	≤0.5	20 (71.40)	54 (66.70)	30 (26.30)	42.53	<0.001
	0.5–1	7 (25.00)	13 (16.00)	36 (28.60)		
	≥1	1 (7.90)	14 (17.23)	48 (42.10)		
Disease etiology	Viral Hepatitis B	12 (42.90)	35 (43.20)	62 (54.40)	8.206^a^	0.405
	Alcoholic Hepatitis	6 (21.40)	16 (19.80)	23 (20.20)		
	Autoimmune Hepatitis	6 (21.40)	8 (9.90)	8 (7.00)		
	Combination of two or more	2 (7.10)	12 (14.80)	13 (11.40)		
	Other	2 (7.10)	10 (12.30)	8 (7.00)		
Cirrhosis staging	Reparative period	1 (3.60)	10 (13.20)	53 (46.50)	36.861	<0.001
	Decompensated period	27 (96.40)	71 (87.70)	61 (53.50)		
Ascites	Yes	17 (60.70)	43 (53.10)	32 (28.10)	17.231	<0.001
	No	11 (39.30)	38 (46.90)	82 (71.90)		
Duration of illness (months/years)	≤6 months	12 (42.90)	35 (43.20)	72 (63.20)	16.792^a^	0.059
	6 months – 1 year	4 (14.30)	5 (6.20)	3 (2.60)		
	1–3 years	5 (17.90)	16 (19.80)	16 (14.00)		
	3–5 years	4 (14.30)	6 (7.40)	4 (3.50)		
	5–10 years	2 (7.10)	12 (14.80)	13 (11.40)		
	≥10 years	1 (3.60)	7 (8.60)	6 (5.30)		
Child-Pugh	A	2 (7.10)	9 (11.10)	45 (39.50)	39.072	<0.001
	B	9 (32.10)	37 (45.70)	50 (43.90)		
	C	17 (60.70)	35 (43.20)	19 (16.70)		
Complications	None	3 (10.70)	9 (11.10)	48 (42.10)	36.827	<0.001
	Combined 1	5 (17.90)	23 (28.40)	33 (28.90)		
	Combined 2	11 (39.30)	26 (32.10)	21 (18.40)		
	Combined 3 or more	9 (32.10)	23 (28.40)	12 (10.50)		
Days of hospitalization (days)	1–10	10 (35.70)	44 (54.30)	78 (68.40)	20.841	<0.001
	10–20	10 (35.70)	30 (37.00)	31 (27.20)		
	≥20	8 (28.60)	7 (8.60)	5 (4.40)		

^a^Denotes Fisher’s exact probability method; USD, United States Dollar (CNY/USD:7.1705).

### Multivariate logistic regression results

To identify factors influencing latent trajectory classes of symptom burden in patients with liver cirrhosis, 10 variables that were statistically significant in univariate analysis—sex, age, educational level, per capita monthly household income, average daily exercise time, stage of liver cirrhosis, presence of ascites, Child–Pugh classification, number of comorbidities, and length of hospital stay—were included in a multivariate logistic regression model. The three latent trajectory classes served as the dependent variable. The overall regression model was statistically significant, with a likelihood ratio χ^2^ = 140.616 (*p* < 0.05). The results indicated that sex, average daily exercise time, and Child–Pugh classification were independent predictors of trajectory membership. Coding of demographic and clinical characteristics is presented in Table 9, and detailed multivariate logistic regression results are shown in Table 10.

**Table 9:** Multiple logistic regression for cirrhosis patients assignment of dependent variables.

Independent variable	Assignment of values
Gender	males = 1; female = 2
Age (years)	18–44 = 1; 45–59 = 3; ≥60 = 3
Educational level	illiteracy = 1; secondary schools = 2; Junior/Secondary = 3; High school/college = 4; University and above = 5
Average monthly income of family members (USD)	≤140;140–418 = 2; ≥418 = 3
Average daily exercise time (h/d)	≤0.5; 0.5–1; ≥1
Cirrhosis stage	Reparative period = 1; Decompensated period = 2
Ascites	Yes = 1; No = 2
Child-Pugh	A = 1; B = 2; C = 3
Number of complications	No = 1; Combined 1 = 2; Combined 2; Combined 3 and more = 3
Number of days of hospitalization (days)	1–10 = 1; 10–20 = 2;≥20

USD, United States Dollar (CNY/USD:7.1705).

**Table 10:** Multivariate logistic regression results for trajectories of symptom burden in patients with liver cirrhosis.

Predictor variable	Categorization	*B*	Wald value	OR	95%CI		*p*
					limit	lower limit	
C2 vs. C3^a^							
Gender	Male	−0.044	0.005	0.957	0.296	3.093	0.942
Average daily exercise time (h/d)	≤0.5	1.049	5.054	2.854	1.144	7.119	0.025
	0.5–1	−0.050	0.009	0.951	0.338	2.680	0.925
Child-Pugh	A	−2.059	7.005	0.128	0.028	0.586	0.008
	B	−0.683	1.854	0.505	0.189	1.350	0.173
C1 vs. C3^a^							
Gender	Male	−1.903	5.061	0.149	0.028	0.783	0.024
Average daily exercise time (h/d)	≤0.5	2.914	5.000	18.431	1.433	237.055	0.025
	0.5–1	2.831	4.199	16.962	1.131	254.359	0.040
Child-Pugh	A	−3.499	6.916	0.030	0.002	0.410	0.009
	B	−1.700	5.290	0.183	0.043	0.778	0.021
C1 vs. C2^b^							
Gender	Male	−1.859	5.889	0.156	0.035	0.699	0.015
Average daily exercise time (h/d)	≤0.5	1.865	2.108	6.459	0.521	80.128	0.147
	0.5–1	2.881	4.331	17.833	1.182	268.941	0.037
Child-Pugh classification	A	−1.441	1.297	0.237	0.020	2.826	0.255
	B	−1.017	2.398	0.362	0.100	1.310	0.122

^a^Indicates the low symptom level decline group as the reference category; ^b^indicates the medium symptom level decline group as the reference category.

## Discussion

This study aimed to investigate the trajectories of symptom burden and their associated factors in patients with liver cirrhosis. The results revealed three heterogeneous trajectory subgroups: a high symptom burden risk group, a moderate symptom-decreasing group, and a low symptom-decreasing group, accounting for 12.56, 36.32, and 51.12% of the sample, respectively. These findings suggest that longitudinal assessment of symptom burden can help identify the patient subgroup at greatest risk of adverse outcomes. The results also underscore the need for early identification of high-risk patients with elevated symptom burden and the timely implementation of targeted, stratified symptom management interventions to alleviate symptoms and improve clinical outcomes and quality of life.

The results of this study indicate that one month after discharge represents a critical turning point for patients with liver cirrhosis to develop membership in the high symptom burden risk group. Patients in this trajectory remain in a persistently high-risk symptom fluctuation state over time, which may be related to the recurrent nature of liver cirrhosis and the associated risk of hospital readmission. Previous studies have reported that 69% of patients with liver cirrhosis experience at least one hospital readmission, with an average interval of 67 days between admissions (25). In addition, the rate of unplanned readmission within 30 days is relatively high, ranging from 17 to 37%. Frequent readmissions are associated with multiple factors, including heterogeneity of etiology, differences in disease severity, occurrence of complications, and medication adherence, and have been shown to be independently correlated with patient mortality. Furthermore, the recurrent nature of the disease contributes to adverse psychological states such as anxiety and depression, with prevalence ranging from 16 to 70% (26, 27). Therefore, close monitoring should be prioritized for patients at high risk of elevated symptom burden, as well as for warning signs of decompensated liver cirrhosis, such as jaundice, ascites, and lower limb edema. Long-term follow-up via telecommunication channels, including WeChat or telephone, can facilitate timely online consultations or outpatient visits for patients with high symptom burden, enabling early detection of symptom deterioration and prompt intervention (28). In addition, providing psychological and social support after discharge is crucial for patients with liver cirrhosis. Psychological assessment at the time of discharge can identify patients experiencing anxiety or depression, allowing for early interventions such as online cognitive behavioral therapy and psychological counseling. Family members can be guided to strengthen emotional support, and peer support groups can help alleviate negative emotions (29, 30), ultimately reducing the likelihood of patients transitioning into the high symptom burden risk group. Previous studies have indicated that ascites and hepatic encephalopathy are the primary causes of hospital readmission in patients with liver cirrhosis (31). This suggests that improving the management of these complications may reduce disease burden and lower readmission rates. Therefore, post-discharge disease management and medication guidance should be strengthened. One-on-one medication education should be provided before discharge, and medication adherence should be assessed within one month after discharge. Patients should be instructed to strictly limit sodium and fluid intake, use diuretics appropriately, and regularly monitor blood ammonia and electrolyte levels to prevent exacerbation of complications. Additionally, dietary guidance tailored to the patient’s disease characteristics should be provided to improve nutritional status (32–34). In this study, the readmission rate among patients with liver cirrhosis was 21.52%, further confirming the recurrent nature of the disease. These findings highlight the importance of close follow-up during the first month after discharge to monitor changes in symptom levels and initiate timely interventions. Notably, approximately 25% of readmission events can be prevented through enhanced outpatient monitoring and optimization of medication regimens, and digital health interventions have the potential to further reduce readmission rates (35, 36). Furthermore, studies have demonstrated that outpatient follow-up management involving nursing staff can reduce the risk of hospital readmission, primarily by providing targeted care and treatment for specific complications, educational support, and guidance for monitoring signs of decompensation through standardized hospital follow-up (37). In the future, nurse-led multidisciplinary follow-up teams could perform joint assessments at the time of discharge, dynamically adjust intervention plans, and provide timely support for patients in the high symptom burden group, thereby reducing readmissions within the first month after discharge. Based on this evidence, future research should focus on developing early intervention mechanisms through multidisciplinary collaboration and establishing multidimensional, comprehensive intervention models. Implementing a standardized follow-up system during the critical first month post-discharge, combined with evidence-based, precision intervention strategies, may effectively lower readmission rates and improve survival and quality of life for patients with liver cirrhosis.

According to the dynamic symptom management model, antecedents of symptoms include patient demographic characteristics, clinical features, individual disease factors, contextual factors, and symptom experiences, while trajectories of symptom changes can facilitate improved symptom assessment and management (38). In this study, multivariate logistic regression analysis of symptom burden trajectories in patients with liver cirrhosis indicated that sex, average daily exercise time, and Child–Pugh classification were significant predictors of symptom trajectory over the 6 months following discharge. Assessing these predictive factors prior to discharge can help identify patients at risk of belonging to the high symptom burden group, enabling timely intervention targeting key determinants and the implementation of tailored symptom management strategies, thereby mitigating the impact of symptom clusters on quality of life.

This study, based on longitudinal data analysis, revealed significant sex-related heterogeneity in trajectories of symptom burden among patients with liver cirrhosis. Compared with male patients, female patients were more likely to belong to the high symptom burden risk group. Previous studies have indicated that female patients with non-alcoholic fatty liver disease are at a higher risk of progressing to advanced fibrosis compared with male patients (39), a finding with significant implications for exploring sex differences in liver disease burden. Recent reports have also shown that the incidence of liver cirrhosis among women has increased more rapidly than in men, with a 12% higher mortality rate in female patients (40), suggesting that women may currently face unique risk factors and increased susceptibility to chronic liver disease. Accordingly, early identification of sex-specific risk factors, reversal of pathogenic mechanisms, prevention of complications, and implementation of gender-tailored interventions are of critical importance. Studies have reported that postmenopausal female patients with liver cirrhosis lose the protective effects of estrogen, which may contribute to continuous disease progression (41). Additionally, female patients with liver cirrhosis are at higher risk of depression and anxiety, with the prevalence of severe depressive and anxiety disorders in women being 1.5 times that of men, potentially related to age composition within the female population (42). Studies have shown that female sex is a significant factor contributing to muscle cramps in patients with chronic liver disease, which may be attributed to lower muscle mass in women (43). Overall, both the prevalence and symptom burden of chronic liver disease exhibit sex-related differences, and future research should further investigate the relationships between sex and symptom trajectories, considering factors such as disease characteristics, hormonal status, and immune function, to validate the findings of the present study (44). In this study, most female patients with liver cirrhosis were from rural areas in China and have long assumed domestic responsibilities, which may increase psychological burden due to economic pressure, prolonged home confinement, and unemployment. Evidence indicates that in western provinces of China, particularly rural areas, mortality among patients with liver cirrhosis is significantly higher than in urban areas, reflecting disparities in healthcare resources and economic development. Advanced age, low socioeconomic status, and financial burden are key factors that reduce patients’ quality of life, and their needs in disease management, psychosocial support, and economic assistance are often unmet (45). Therefore, greater healthcare attention, psychological support, and comprehensive interventions to enhance self-management skills should be provided for rural female patients with liver cirrhosis, which is critical to preventing progression into the high symptom burden risk group.

In this study, patients with liver cirrhosis who engaged in less than one hour of daily physical activity were at significantly higher risk of belonging to the moderate symptom-decreasing and high symptom burden risk groups, consistent with existing evidence. Previous research has shown that 76% of the waking time of patients with liver cirrhosis is spent in sedentary behavior, which is a risk factor for fibrosis progression in chronic liver disease (46). Multiple randomized controlled trials have demonstrated the beneficial effects of exercise on various chronic diseases, and structured exercise training has been shown to improve the health status of patients with liver cirrhosis. Specifically, 8–14 weeks of sustained aerobic or resistance exercise can enhance cardiopulmonary function, muscle strength, and quality of life while reducing the risk of hepatic decompensation (47–49). Furthermore, interventions combining moderate-intensity exercise with nutritional support have been shown to prevent the development of hepatic encephalopathy in patients with liver cirrhosis (50). Within the limits of patient condition, a combination of aerobic and resistance training, performed at least 3 days per week for 30–60 min per session at moderate intensity under supervision, has demonstrated significant benefits for complications and prognosis in patients with liver cirrhosis (51). The findings of this study indicate that physical activity can improve symptom levels; however, daily exercise time was obtained through patient recall during interviews, which may introduce some recall bias. This highlights the need for future studies to adopt more objective monitoring methods to accurately quantify exercise dose. Objective monitoring is crucial for guiding therapy in patients with liver cirrhosis, and technologies such as wearable activity trackers or virtual reality can be utilized for home-based telehealth or virtual interventions to effectively monitor and assess post-discharge activity levels (52, 53). At present, exercise interventions for patients with liver cirrhosis remain in the developmental stage. Home-based exercise and encouragement of non-exercise physical activities are recommended to promote sustained increases in activity levels (54). Therefore, structured and rational exercise interventions represent an important component of liver cirrhosis management. Healthcare providers should leverage multidisciplinary teams and telemedicine technologies to deliver appropriate health monitoring and ensure the safety and effectiveness of exercise programs. Future studies should focus on investigating the effects of different exercise modalities, intensities, frequencies, and durations on symptom improvement in patients with liver cirrhosis, thereby providing more robust evidence-based guidance for clinical exercise prescription.

Multivariate logistic regression analysis in this study demonstrated that patients with Child–Pugh class C cirrhosis were at significantly higher risk of belonging to the high symptom burden risk group compared with those with class A or B. The Child–Pugh scoring system, which integrates key parameters including serum total bilirubin, albumin, ascites severity, hepatic encephalopathy grade, and prothrombin time, has been widely validated as an effective tool for assessing cirrhosis severity and prognostic risk (55). Existing evidence indicates that the Child–Pugh score is significantly positively correlated with patient mortality and can accurately predict the risk of complications related to hepatic decompensation (56). In this study, most patients with Child–Pugh class C cirrhosis were in the decompensated stage, presenting with severe clinical features such as refractory ascites, gastrointestinal bleeding, jaundice, and generalized edema. These patients not only experience prolonged treatment courses but also have markedly higher symptom recurrence, which can undermine confidence in treatment and daily life, leading to negative psychological states. Consequently, they are more likely to belong to the high symptom burden risk group (57). It is noteworthy that significant differences in Child–Pugh class and cirrhosis stage were observed between the follow-up and lost-to-follow-up groups in this study. Among patients lost to follow-up, 66.7% were classified as Child–Pugh class C, 86.7% were in the decompensated stage, and 62.2% were lost due to death, which is directly related to higher symptom burden and poorer prognosis in this population. This indicates the presence of attrition bias in the study. If these severely ill patients had completed the full follow-up, the actual prevalence of the high symptom burden risk group might exceed the 12.56% observed, and symptom trajectory changes could be more pronounced. Therefore, the findings are more applicable to patients with stable disease and survival exceeding 6 months. Nevertheless, this observation also indirectly supports that Child–Pugh class C is a key predictor of poor outcomes, including high symptom burden and mortality, thereby reinforcing the reliability of the study conclusions. Based on these insights, future studies should implement proactive follow-up for decompensated patients, establish a dynamic monitoring system for liver function and symptom assessment in Child–Pugh class C patients, and provide multidisciplinary early interventions to reduce high symptom burden risk. Additionally, propensity score matching should be applied to balance clinical characteristics between groups, minimize attrition bias, accurately estimate the true prevalence of the high symptom burden risk group, and explore the differential effects of individual Child–Pugh score components on symptom trajectories, thereby providing more robust evidence for precision symptom management in patients with decompensated cirrhosis.

This study innovatively applied a latent class growth model (LCGM) to elucidate the trajectories of symptom burden and their heterogeneous influencing factors in patients with liver cirrhosis, achieving, for the first time, precise identification of high-risk intervention groups and critical time points, particularly one month post-discharge. Healthcare professionals should promptly assess the intervention targets in high-risk cirrhosis patients and implement early measures to alleviate symptom burden, thereby improving clinical outcomes and quality of life, delaying disease progression, enhancing survival, and reducing readmission rates, which also facilitates optimized allocation of medical resources. Furthermore, in the development of dynamic symptom management programs, the findings of this study enrich longitudinal research on symptom trajectories in liver cirrhosis, provide objective and scientific evidence for precision symptom management strategies, and offer valuable insights for dynamic assessment of symptom burden in other chronic diseases.

### Innovative contributions

This pioneering study employed a latent class growth model (LCGM) to statistically model the trajectories of symptom burden in patients with liver cirrhosis, identifying the heterogeneity of longitudinal symptom changes and their influencing factors. The study provides threefold clinical significance: (1) offering evidence to support stage-specific precision interventions; (2) establishing a theoretical basis for evidence-based identification of high-risk populations and the definition of critical intervention time windows; and (3) advancing clinical validation of symptom burden trajectories in cirrhosis and promoting a paradigm shift in symptom research toward temporal sequencing.

### Limitations

This study has several limitations. First, it was a single-center convenience sampling study conducted in a tertiary hospital in Zunyi. Restricted by the single geographical origin and clinical diagnosis and treatment background of the study subjects, as well as the geographical and socioeconomic characteristics of Southwest China, the generalizability of the study results is limited. In the future, our team will collaborate with multiple tertiary hospitals inside and outside the province to conduct multi-center, large-sample, randomized controlled studies. We suggest that healthcare workers in different geographical regions and clinical settings conduct small-sample local validation before applying the results of this study, so as to enhance the external validity and clinical universality of the results. Second, data were collected via telephone and WeChat, which may introduce reporting bias, and follow-up was limited to 6 months post-discharge, precluding observation of long-term symptom trajectories for some patients. Moreover, attrition bias, the lack of covariate analysis, and potential residual confounding in multivariable regression limit the ability to explore dynamic interactions among multiple factors, resulting in slightly reduced statistical power. Future studies should expand the geographical scope, implement long-term longitudinal assessments, and apply appropriate statistical methods and more powerful multivariable analytical approaches to further validate the long-term effects and applicability of these findings.

## Conclusion

This study identified heterogeneous trajectories of symptom burden in patients with liver cirrhosis using a latent class growth model, with one month post-discharge representing a critical turning point for the development of the high symptom burden risk group. These findings suggest that healthcare professionals should proactively predict the trajectory patterns of high-risk patients and prioritize intervention targets, including gender (female), average daily physical activity (<1 h/day), and Child–Pugh class C, to inform the implementation of precision stratified interventions. Future efforts may focus on establishing an intelligent, multidisciplinary, time-sequenced dynamic monitoring system to alleviate symptom burden, improve adverse clinical outcomes, and enhance quality of life.

## Data Availability

The original contributions presented in the study are included in the article/supplementary material, further inquiries can be directed to the corresponding author.
